# Indigenous Wildlife Rabies in Taiwan: Ferret Badgers, a Long Term Terrestrial Reservoir

**DOI:** 10.1155/2017/5491640

**Published:** 2017-04-12

**Authors:** Yu-Ching Lan, Tzai-Hung Wen, Chao-chin Chang, Hsin-Fu Liu, Pei-Fen Lee, Chung-Yuan Huang, Bruno B. Chomel, Yi-Ming A. Chen

**Affiliations:** ^1^Department of Health Risk Management, China Medical University, Taichung, Taiwan; ^2^Department of Geography, National Taiwan University, Taipei, Taiwan; ^3^Graduate Institute of Microbiology and Public Health, School of Veterinary Medicine, National Chung Hsing University, Taichung, Taiwan; ^4^Department of Medical Research, Mackay Memorial Hospital, Taipei, Taiwan; ^5^Institute of Ecology and Evolutionary Biology, National Taiwan University, Taipei, Taiwan; ^6^Department of Computer Science and Information Engineering, Chang Gung University, Taoyuan, Taiwan; ^7^Department of Population Health and Reproduction, School of Veterinary Medicine, University of California, Davis, Davis, CA, USA; ^8^Department of Microbiology, School of Medicine, Kaohsiung Medical University, Kaohsiung, Taiwan

## Abstract

The emerging disease of rabies was confirmed in Taiwan ferret badgers (FBs) and reported to the World Organization for Animal Health (OIE) on July 17, 2013. The spread of wildlife rabies can be related to neighborhood countries in Asia. The phylogenetic analysis was conducted by maximum likelihood (ML) methods and the Bayesian coalescent approach based on the glycoprotein (G) and nucleoprotein (N) genes. The phylogeographic and spatial temporal dynamics of viral transmission were determined by using SPREAD, QGIS. Therefore, the origin and the change with time of the viruses can be identified. Results showed the rabies virus of FB strains in Taiwan is a unique clade among other strains in Asia. According to the phylogeographic coalescent tree, three major genotypes of the FB rabies virus have circulated in three different geographical areas in Taiwan. Two genotypes have distributed into central and southern Taiwan between two ecological river barriers. The third genotype has been limited in southeastern Taiwan by the natural mountain barrier. The diversity of FB rabies viruses indicates that the biological profile of FBs could vary in different geographical areas in Taiwan. An enhanced surveillance system needs to be established near the currently identified natural barriers for early warnings of the rabies virus outbreak in Taiwan.

## 1. Introduction

Taiwan had been recognized as a rabies-free area since 1961 (http://www.nvri.gov.tw/index.aspx). However, in June 2013, after ruling out the possibility of canine distemper and pseudorabies infections in three dead ferret badgers* (Melogale moschata)* from Nantou and Yunlin Counties and using a series of tests for rabies diagnosis at the National Taiwan University, including Immunohistochemistry (IHC) and RT-PCR, the first rabies case was finally confirmed on June 26, 2013 by the National Laboratory, Animal Health Research Institute, Council of Agriculture, and Executive Yuan, Taiwan. The results were reported to the World Organization for Animal Health (OIE) on July 17, 2013, and Taiwan was declared as a rabies-infected area.

Since then, several public health measures have been applied for preventing rabies transmission from animals to humans [1]. Those strategies were including enhanced surveillance for human rabies cases by testing stored cerebrospinal fluids (CSF) from patients with encephalitides of unknown cause by RT-PCR. The prioritizing vaccine has been used for postexposure prophylaxis (PEP) during periods of vaccine shortage. The expansion of PEP use and surveillance of animal bites, surveillance for adverse events following immunization (AEFI), health education for general population, and also healthcare professionals had been provided for rabies control by various Taiwan public health systems [1].

A variety of island-wide wild species especially carnivores have been reported exhibiting aggressive behaviors, neurologically ill, and dead. Among more than hundreds of carnivores tested so far for rabies, confirmed rabies cases were almost exclusively (99%) identified in ferret badgers (FBs). In a surveillance report for 1016 wild carnivores, 33.2% from 831 Formosan FBs were fluorescent antibody test (FAT) positive. Most of other animals tested, including dogs, cats, bats, mice, house shrews, and squirrels, were rabies-negative [2]. However, there were two rabies cases identified in other animal species, including one 1.5-month-old dog after bitten by a rabid FB and one shrew (*Suncus murinus*). Although several wildlife species have been shown to be the main reservoirs of rabies around the world, the role of FBs in rabies transmission to dogs and humans has been identified only in Mainland China [3].

Although a surveillance system for canine rabies has been established in Taiwan for many years, the unique rabies viral strains found in FBs in this study seem to have been silently circulating in this animal species but without spillover to domestic dogs or cats in Taiwan for a long period of time. How this rabies virus strain was introduced in Taiwan and the main reason for the emergence of this outbreak are still unknown and need further investigation. This incidental discovery of wildlife rabies in Taiwan raised two major questions: was the emergence of wildlife rabies due to the accidental introduction of the virus through illegal animal importation from neighborhood countries in Asia? Or was the disease endemic in FBs but went undetected since dog rabies was eradicated in Taiwan? This study was thus conducted to address these issues.

## 2. Materials and Methods

**Figure 1:**
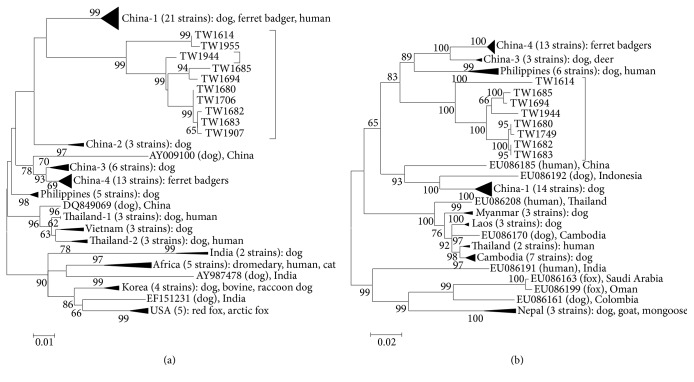
Phylogenetic analysis of rabies virus strains identified in Asia using amino acid sequences. (a) Maximum likelihood (ML) tree based on glycoprotein sequences; (b) maximum likelihood (ML) tree based on nucleoprotein sequences.

**Figure 2:**
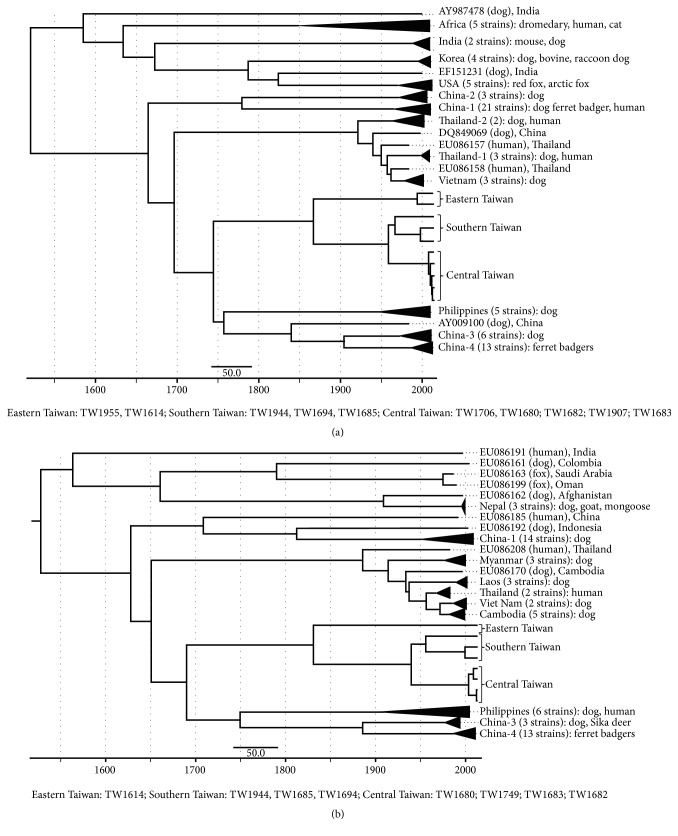
Coalescent analysis of rabies virus strains identified in Asia. (a) Using amino acid sequences of glycoprotein; (b) using amino acid sequences of nucleoprotein.

Phylogenetic analysis by maximum likelihood (ML) methods and coalescent analysis (Figures 1 and 2) were applied to identify the possible origin of this rabies outbreak by using glycoprotein sequences of 10 viral strains and nucleoprotein sequences of 8 viral strains isolated from FBs in Taiwan (http://www.nvri.gov.tw/Module/NewsContent/400/392.aspx?nid=%2bp7kpxBWWe0%3d&type=zO4l76wykT8%3d; 2013/11/15; GenBank Accession number: KF501174.1-KF501185.1). These sequences were compared to sequences of 75 glycoprotein and 63 nucleoprotein of rabies viral strains in other countries, including China, Philippines, Thailand, Vietnam, India, Africa Korea, Laos, Cambodia, Nepal, and USA (please refer to supplemental Tables 1 and 2 in Supplementary Material available online at https://doi.org/10.1155/2017/5491640 for detailed information of the viral strains).

The software of MEGA version 5.2 (http://www.megasoftware.net/) was used for ML analysis. The maximum clade credibility (MCC) tree of these two proteins was constructed by BEAST version 1.7.5 (http://beast.bio.ed.ac.uk), which implements a Bayesian coalescent approach through Markov-Chain Monte Carlo method for 80 million samplings. The estimated time to most recent common ancestor (TMRCA) and its 95% highest posterior density (HPD) values were obtained.

The phylogeographic tree by SPREAD 1.0.6 (http://www.phylogeography.org/SPREAD.html) and google earth ArcGIS was constructed for understanding if the viral evolution could be associated with ecological barriers. Posterior distributions under the Bayesian phylogeographic model [4] were estimated using a MCMC method implemented in BEAST. For all animals with rabies reported by Taiwan Bureau of Animal and Plant Health Inspection and Quarantine, the geographic locations were integrated into QGIS 2.1.8.

## 3. Results

**Figure 3:**
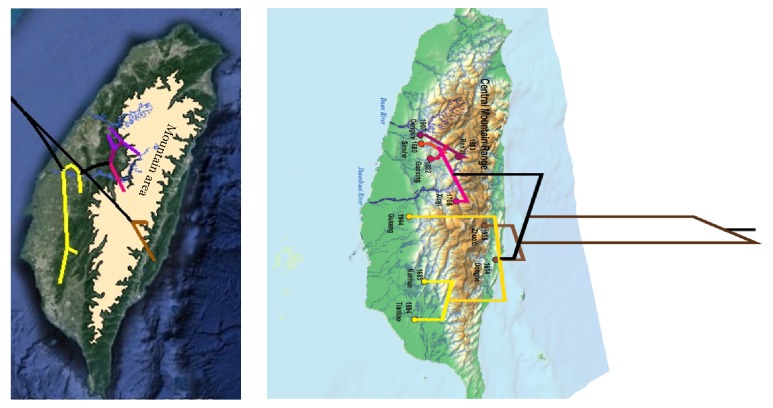
Phylogeographic analysis of three genotypes of rabies virus strains in ferret badgers in Taiwan at google earth (figure on the left). The figure on the right shows the details about the location. Three genotypes identified in central, southern, and southeastern Taiwan were separated by three ecological barriers, the Da-An River, the JhuoShuei River, and the Central Mountain Range.

In comparison with the rabies viral strains in southeastern Asia, the FB strains in Taiwan were shown as a unique clade (Figures 1 and 2). The FB rabies virus strains in Taiwan originated much earlier (median: 1870; 95% HPD: 1757~1946) than the FB rabies virus strains in Mainland China (median: 1987; 95% HPD: 1970~1999). The phytogeographic tree and its clear geographical details (Figure 3) further indicated that there were three major genotypes of the FB rabies viruses circulating in three different geographical areas in Taiwan. The earliest virus isolation may have emerged by 1965 (median = 1965; 95% HPD: 1942~1982) in southern Taiwan, and the most recent virus may have emerged in 2008 (median: 2008; 95% HPD: 2004~2011) in central Taiwan (glycoprotein).

As shown in Figure 3, the two genotypes identified in central and southern Taiwan were separated by two ecological barriers, the Da-An River and the JhuoShuei River. These two rivers are among the main river systems in Taiwan. The width of these rivers may constrain the distribution of these animals. Another genotype was further identified in southeastern Taiwan, which could have emerged at the end of the 20th Century (median: 1993; 95% HPD: 1980~2002). The spreading of this genotype from the east to the west in Taiwan may be limited by the natural barrier, the Central Mountain Range. The Central Mountain Range with high elevation running from the north to the south of the main island is one of the five principal mountain ranges in Taiwan. Our results further indicated that the substitution rates of FB rabies viruses were 0.02, 0.022, and 0.015 in central, southern, and eastern Taiwan, respectively.

**Figure 4:**
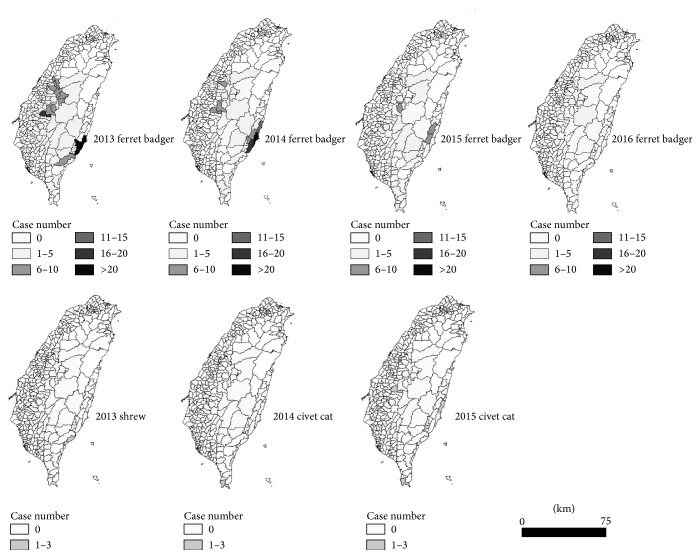
The number of animals with rabies viruses had been reported by Taiwan Bureau of Animal and Plant Health Inspection and Quarantine between 2013 and 2016. The major species were FBs and others were civet cat, shrew, and dog.

According to the report from Bureau of Animal and Plant Health Inspection and Quarantine in Taiwan between 2013 and 2016, there was one imported human case from other countries at 2013. Among 556 animals with rabies, there were 548 FBs (98.6%), 6 civet cat (1.1%), 1 shrew, and 1 dog. The geographic distribution of those animals with Taiwan rabies viruses had sporadically shown the high mountain areas still had few infected shrews, FBs, and also civet cats (Figure 4). Most of the cases were still FBs. Other animals, such as shrew and civet cats, had not been limited by FBs' geographical barriers. Furthermore, the infected numbers decreased from 2013 to 2016.

## 4. Discussion

Our results further indicated that the substitution rates of FB rabies viruses were similar in central and southern Taiwan. The observations may imply a similar ancestral history of rabies virus lineages in central and southern Taiwan. After the rabies virus adapted to a new host species, rates of viral evolution may be more likely affected by biology of the maintenance host rather than rabies virus itself [5]. Therefore, the diversity of FB rabies viruses circulating in Taiwan may suggest that the biological profile of FBs, a potential factor to drive rabies virus evolution, could vary in different geographical areas in Taiwan. Our results also indicate that an enhanced surveillance system needs to be established on appropriate sites near the currently identified natural barriers for early warning of possible dispersion of rabies viruses to new areas.

The long term existence and unique strains of FB rabies viruses found in different geographical areas in Taiwan, which is similar with previous studies [2, 6, 7], may rule out the possibility of recent virus introduction through illegal importations of animals, such as wild ferret badgers, from nearby rabies-endemic countries. A study in domestic ferrets* (Mustela putorius furo)* showed a low isolation rate of rabies virus from the salivary glands of the ferrets after inoculation of rabies viral strains from naturally infected ferrets [8]. For efficient rabies transmission among animals, the viral load needs to be high enough in the saliva. The recent study performed the pathologic and molecular detection of rabies virus in three Taiwan FBs and found that the earliest infected case could be traced back to 2004. In these FBs nonnervous system tissues, representative lesions included adrenal necrosis and lymphocytic interstitial sialadenitis [9]. The higher seroprevalence of rabies infection in FBs reported in Mainland China [3] also implied that FBs may be less capable reservoir animals in comparison to other wild carnivores for rabies transmission. Therefore, similar studies need to be conducted in FBs in Taiwan to understand why no other wild mammal or even dog rabies cases were reported in the epizootic areas before identification of this outbreak.

The results shown by the present phylogeographic analysis support the idea that the three unique rabies viral genotypes circulating in FBs could be related to geographical barriers in Taiwan. The similar findings have been shown in previous studies to understand evolution of dog rabies viruses [2, 10]. After confirming the broad-scale observations from the phylogenetic analysis among dog rabies, it revealed a strong population subdivision by geographical region among dogs in Africa, Asia, America, and Europe [10]. Across 130 sequences from 17 geographical regions' dog, researchers just found only 17 migration events. Most of the migration events involved contiguous geographical regions, with only occasional long distance migration. The researchers suggested that some of the migrations are probably due to human interventions.

Taiwan rabies from FBs also presented the division by geographical region [2, 7]. The previous phylogeographic analysis suggested a similar conclusion with this study; the Formosan FBs moved across the Central Range of Taiwan and separated into two branches [7]. The geographic information evidence (Figure 4) presented the notion that the infected animals have sporadically been found in the high mountain areas which shows the probability of how these animals carried the viruses and climbed through the mountain. The correlation of these infected animals in the mountain and human behavior need to be identified in the future.

Moreover, ecological studies on population density, seasonal movement, and eating behaviors of FBs in Taiwan are necessary to understand the implications of these factors for rabies transmission among these animals, the interaction of FBs, and other kinds of wildlife living in the same environment. This information will be helpful to establish a more efficient surveillance system for understanding the dynamic changes of rabies epidemics in Taiwan. Also, the results can be applied for the development of effective oral rabies vaccines for rabies control among wild ferret badgers in Taiwan. Moreover, the infectious case of rabies virus in human has not been found in Taiwan area. Therefore, further virulence testing is suggested for the degree of cross-species transmission.

## Supplementary Material

The background information of glycoprotein (G) and nucleoprotein (N) reference sequences of the rabies viral strains using in this study for phylogenetic data analysis presented the GenBnak accession number, country source and the year of isolation. The 75 glycoprotein and 63 nucleoprotein of rabies viral strains came from China, Philippines, Thailand, Vietnam, India, Africa Korea, Laos, Cambodia, Nepal, and USA.

## References

[1] Huang A. S.-E., Chen W.-C., Huang W.-T. (2015). Public health responses to reemergence of animal rabies, Taiwan, July 16-December 28, 2013. *PLoS ONE*.

[2] Tsai K. J., Hsu W. C., Chuang W. C. (2016). Emergence of a sylvatic enzootic formosan ferret badger-associated rabies in Taiwan and the geographical separation of two phylogenetic groups of rabies viruses. *Veterinary Microbiology*.

[3] Liu Y., Zhang S., Wu X. (2010). Ferret badger rabies origin and its revisited importance as potential source of rabies transmission in Southeast China. *BMC Infectious Diseases*.

[4] Lemey P., Rambaut A., Drummond A. J., Suchard M. A. (2009). Bayesian phylogeography finds its roots. *PLoS Computational Biology*.

[5] Streicker D. G., Lemey P., Velasco-Villa A., Rupprecht C. E. (2012). Rates of viral evolution are linked to host geography in bat rabies. *PLoS Pathogens*.

[6] Chiou H.-Y., Hsieh C.-H., Jeng C.-R., Chan F.-T., Wang H.-Y., Pang V. F. (2014). Molecular characterization of cryptically circulating rabies virus from ferret badgers, Taiwan. *Emerging Infectious Diseases*.

[7] Lin Y.-C., Chu P.-Y., Chang M.-Y., Hsiao K.-L., Lin J.-H., Liu H.-F. (2016). Spatial temporal dynamics and molecular evolution of re-emerging rabies virus in Taiwan. *International Journal of Molecular Sciences*.

[8] Niezgoda M., Briggs D. J., Shaddock J., Dreesen D. W., Rupprecht C. E. (1997). Pathogenesis of experimentally induced rabies in domestic ferrets. *American Journal of Veterinary Research*.

[9] Chiou H.-Y., Jeng C.-R., Wang H.-Y. (2016). Pathology and molecular detection of rabies virus in ferret badgers associated with a rabies outbreak in Taiwan. *Journal of Wildlife Diseases*.

[10] Bourhy H., Reynes J.-M., Dunham E. J. (2008). The origin and phylogeography of dog rabies virus. *Journal of General Virology*.

