# Supplementary material for: Indigenous Wildlife Rabies in Taiwan: Ferret Badgers, a Long Term Terrestrial Reservoir
Source: Biomed Res Int. 2017 Apr 12;2017:5491640. doi: 10.1155/2017/5491640 (PMC5405374; doi:10.1155/2017/5491640)
Supplement: Supplementary file 1 — The background information of glycoprotein (G) and nucleoprotein (N) reference sequences of the rabies viral strains using in this study for phylogenetic data analysis presented the GenBnak accession number, country source and the year of isolation. The 75 glycoprotein and 63 nucleoprotein of rabies viral strains came from China, Philippines, Thailand, Vietnam, India, Africa Korea, Laos, Cambodia, Nepal, and USA. [file 5491640.f1.docx]

**Supplemental Table 1.Background information of glycoprotein (G) sequences of the rabies virus strains in this study.**

| **Clade/isolate** | **Genbank**  **Accession no.** | **Source** | **Country** | **Year of isolation** |
| --- | --- | --- | --- | --- |
| China-1 | DQ849049 | Dog | China | 2004 |
|  | EU275241 | Dog |  | 2007 |
|  | GU186392 | Dog |  | 2008 |
|  | GQ857462 | Dog |  | 2009 |
|  | DQ849047 | Dog |  | 2004 |
|  | HQ450385 | Dog |  | 2008 |
|  | EU275240 | Dog |  | 2007 |
|  | JQ699232 | Dog |  | 2008 |
|  | GU186382 | Dog |  | 2007 |
|  | GQ857470 | Dog |  | 2006 |
|  | FJ602457 | Dog |  | 2007 |
|  | FJ825127 | Dog |  | 2008 |
|  | GQ857466 | Dog |  | 2008 |
|  | EU828656 | Dog |  | 2007 |
|  | EU086143 | Dog |  | 1989 |
|  | DQ849044 | Dog |  | 2004 |
|  | GU233763 | Ferret badger |  | 2009 |
|  | DQ849064 | Dog |  | 2004 |
|  | DQ849063 | Human |  | 2006 |
|  | DQ849061 | Dog |  | 2005 |
|  | EU700030 | Human |  | 2008 |
|  |  |  |  |  |
| China-2 | GU186387 | Dog | China | 2005 |
|  | GU186396 | Dog |  | 2005 |
|  | DQ849072 | Dog |  | 1992 |
|  |  |  |  |  |
| China-3 | JQ699270 | Dog | China | 2005 |
|  | EU828654 | Dog |  | 2007 |
|  | JQ699262 | Dog |  | 2010 |
|  | JQ699265 | Dog |  | 2008 |
|  | JN936711 | Dog |  | 2006 |
|  | GU186379 | Dog |  | 2005 |
|  |  |  |  |  |
| China-4 | GQ857468 | Ferret badger | China | 2008 |
|  | JN936788 | Ferret badger |  | 2008 |
|  | FJ825135 | Ferret badger |  | 2008 |
|  | GU647092 | Ferret badger |  | 2008 |
|  | FJ719752 | Ferret badger |  | 2008 |
|  | JQ950453 | Ferret badger |  | 2012 |
|  | FJ719749 | Ferret badger |  | 2008 |
|  | JQ950451 | Ferret badger |  | 2012 |
|  | JQ950449 | Ferret badger |  | 2012 |
|  | FJ712196 | Ferret badger |  | 2008 |
|  | FJ712195 | Ferret badger |  | 2008 |
|  |  |  |  |  |
| Philippines | AB563888 | Dog | Philippines | 2008 |
|  | AB563824 | Dog |  | 2006 |
|  | AB683584 | Dog |  | 2004 |
|  | AB563896 | Dog |  | 2008 |
|  | AB564000 | Dog |  | 2009 |
|  |  |  |  |  |
| Korea | GU937029 | Dog | South Korea | 2008 |
|  | GU937030 | Cattle |  | 2009 |
|  | GU937026 | Racoon dog |  | 2008 |
|  | GU937025 | Racoon dog |  | 2009 |
|  |  |  |  |  |
| USA | KC792186 | Red fox | USA | 2010 |
|  | KC792187 | Red fox |  | 2010 |
|  | KC792180 | Arctic fox |  | 2011 |
|  | KC792183 | Arctic fox |  | 2011 |
|  | KC792188 | Arctic fox |  | 2010 |
|  |  |  |  |  |
| Thailand-1 | GQ303558 | Dog | Thailand | 2009 |
|  | HQ166186 | Dog |  | 2000 |
|  | GQ303557 | Human |  | 2009 |
|  |  |  |  |  |
| Thailand-2 | HQ232301 | Dog | Thailand | 2002 |
|  | EU086157 | Human |  | 1983 |
|  | AF325488 | Human |  | 1983 |
|  |  |  |  |  |
| India | GQ233040 | Dog | India | 2001 |
|  | FJ979833 | mouse |  | 2008 |
|  |  |  |  |  |
|  |  |  |  |  |
| Africa | FJ545682 | Dromedary | Africa | 1991 |
|  | FJ545661 | Human |  | 2001 |
|  | FJ545665 | Human |  | 2007 |
|  | FJ545660 | Cat |  | 1997 |
|  | FJ545673 | Cat |  | 1986 |
|  |  |  |  |  |
| Viet Nam | EU086160 | Dog | Viet Nam | 2001 |
|  | EU086159 | Dog |  | 2001 |
|  | EU086158 | Dog |  | 1983 |
|  |  |  |  |  |
| Others | DQ849069 | Dog | China: Guangxi | 1997 |
|  | AY009100 | Dog | China: Shandong | 1983 |
|  | AY987478 | Dog | India: Chandigarch | 1999 |
|  | EF151231 | Dog | India: Bareilly | 1999 |

**Supplemental Table 2.Background information of nucleoprotein (N) sequences of the rabies virus strainsin this study.**

| **Clade/ isolate** | **Genbank**  **Accession no.** | **Source** | **Country** | **Year of isolation** |
| --- | --- | --- | --- | --- |
| China-1 | EU086186 | Dog | China | 2005 |
|  | EU086187 | Dog |  | 2004 |
|  | EU086188 | Dog |  | 2004 |
|  | EU086189 | Dog |  | 2004 |
|  | EU086190 | Dog |  | 2005 |
|  | EU086173 | Dog |  | 1998 |
|  | EU086174 | Dog |  | 1997 |
|  | EU086175 | Dog |  | 1997 |
|  | EU086177 | Dog |  | 1987 |
|  | EU086178 | Dog |  | 1969 |
|  | EU086179 | Dog |  | 1989 |
|  | EU086180 | Dog |  | 1990 |
|  | EU086181 | Dog |  | 1989 |
|  | GU233765 | Ferret badger |  | 2009 |
|  |  |  |  |  |
| China-3 | EU086182 | Dog | China | 1994 |
|  | EU086183 | Dog |  | 1994 |
|  | EU086184 | Sika deer |  | 1993 |
|  |  |  |  |  |
| China-4 | HQ118114 | Ferret badger | China | 2008 |
|  | HQ118115 | Ferret badger |  | 2008 |
|  | HQ118116 | Ferret badger |  | 2008 |
|  | HQ118117 | Ferret badger |  | 2008 |
|  | HQ118118 | Ferret badger |  | 2008 |
|  | JN974877 | Ferret badger |  | 2008 |
|  | FJ598135 | Ferret badger |  | 2008 |
|  | JQ950448 | Ferret badger |  | 2012 |
|  | JQ950450 | Ferret badger |  | 2012 |
|  | JQ950452 | Ferret badger |  | 2012 |
|  | FJ719751 | Ferret badger |  | 2008 |
|  | FJ719753 | Ferret badger |  | 2008 |
|  | FJ719755 | Ferret badger |  | 2008 |
|  |  |  |  |  |
| Philippines | EU086200 | Dog | Philippines | 1994 |
|  | EU086201 | Dog |  | 1995 |
|  | EU086202 | Dog |  | 1994 |
|  | EU086203 | Human |  | 2000 |
|  | EU086204 | Human |  | 2001 |
|  | EU086205 | Human |  | 2004 |
|  |  |  |  |  |
| Cambodia | EU086167 | Dog | Cambodia | 1999 |
|  | EU086168 | Dog |  | 1998 |
|  | EU086169 | Dog |  | 1998 |
|  | EU086171 | Dog |  | 1999 |
|  | EU086172 | Dog |  | 1998 |
|  |  |  |  |  |
| Thailand | EU086206 | Human | Thailand | 1983 |
|  | EU086207 | Human |  | 1983 |
|  |  |  |  |  |
| Viet Nam | EU086209 | Dog | Viet Nam | 2001 |
|  | EU086210 | Dog |  | 2001 |
|  |  |  |  |  |
| Nepal | EU086196 | Dog | Nepal | 1998 |
|  | EU086197 | Goat |  | 1998 |
|  | EU086198 | Mongoose |  | 1998 |
|  |  |  |  |  |
| Myanmar | EU086164 | Dog | Myanmar | 1999 |
|  | EU086165 | Dog |  | 2000 |
|  | EU086166 | Dog |  | 1999 |
|  |  |  |  |  |
| Laos | EU086193 | Dog | Laos | 1999 |
|  | EU086194 | Dog |  | 2002 |
|  | EU086195 | Dog |  | 2002 |
|  |  |  |  |  |
| Others | GU233765 | Ferret badger | China | 2009 |
|  | EU086185 | Human | China | 1992 |
|  | EU086208 | Human | Thailand | 1983 |
|  | EU086191 | Human | India | 1997 |
|  | EU086192 | Dog | Indonesia | 2003 |
|  | EU086161 | Dog | Colombia | 2004 |
|  | EU086170 | Dog | Cambodia | 1997 |
|  | EU086199 | Fox | Oman | 1990 |
|  | EU086163 | Fox | Saudi Arabia | 1987 |
|  | EU086162 | Dog | Afghanistan | 1996 |
